# Metals and Breast Cancer: Risk Factors or Healing Agents?

**DOI:** 10.1155/2011/159619

**Published:** 2011-07-24

**Authors:** Ana-Maria Florea, Dietrich Büsselberg

**Affiliations:** ^1^Department of Neuropathology, Heinrich-Heine University, 40225 Düsseldorf, Germany; ^2^Weill Cornell Medical College in Qatar, Qatar Foundation, Education City, P.O. Box 24144, Doha, Qatar

## Abstract

Metals and metal compounds are part of our environment. Several metals are essential for physiological functions (e.g., zinc or magnesium); while the beneficial effects of others are uncertain (e.g., manganese), some metals are proven to be toxic (e.g., mercury, lead). Additionally there are organic metal compounds; some of them are extremely toxic (e.g., trimethyltin, methylmercury), but there is very little knowledge available how they are handled by organisms. Scientific evidence indicates that long-term exposure to (some) metallic compounds induces different forms of cancer, including breast cancer. On the other side, several metal compounds have clinical use in treating life-threatening diseases such as cancer. In this paper we discuss the recent literature that shows a correlation between metal exposure and breast cancer.

## 1. Introduction

According to the World Health Organization, breast cancer accounts for 16% of all types of cancer deaths globally (total deaths of cancer 7,600,000, total breast cancer deaths 460,000 [1]). It is the most common solid tumor diagnosed in women [2]. Although the incidence of breast cancer increases with age [3], certain lifestyle and environmental factors play an important role on breast cancer risk [4]. Such risk factors include the genetic background and environmental factors. For example, women who have inherited mutations in the BRCA1 or BRCA2 genes have substantially elevated risks of breast cancer [5].

Also an elevated lifetime estrogen exposure might be major risk factor for breast cancer [6]. However, the activation of estrogen receptors alone is not sufficient for the development of breast cancer [7] indicating that other factors play an important role in carcinogenesis. The underlining mechanism could rely on the ability of estrogen and estrogen metabolites to generate reactive oxygen species which induce DNA synthesis, increased phosphorylation of kinases, and activation of transcription factors, such as AP-1, NRF1, E2F, NF-*κ*B, and CREB responsive to either oxidants (e.g., toxins, including metal compounds) or estrogen. Therefore, the genomic instability increases while the activation of transcription factors plays an important role in cell transformation, cell cycle, migration, and invasion [7].

Environmental factors also play a decisive role in breast carcinogenesis together with life-long dietary habits [4]. More and more evidence underlines that external factors are involved in the development of breast cancer: nutrition (obesity and alcohol consumption), smoking, and exposure to carcinogens (e.g., metal compounds) [4]. Multiple reports show that metallic compounds could function as estrogen disruptors [8], while other studies underline the connection between the exposure to metals or metal compounds and breast cancer risk [2, 9, 10].

The present paper discusses emerging data in support of the role of metal compounds in the development of breast cancer. It is envisioned that estrogen-induced metal-mediated signaling is a key complementary mechanism that drives the carcinogenesis process. On the other hand it also highlighted the beneficial effects of metal-derived compounds, which are used for the treatment of cancer (e.g., platinum compounds have been a breakthrough for the treatment of breast cancer) [11].

In the following sections the association of specific metals (and their compounds) in regard to their effects in inducing breast cancer are discussed as well as beneficial effects of metals in treating the same cancer.

## 2. Epidemiologic Studies Illustrating the Effects of Multiple Metals

A large number of epidemiologic studies associate potential risk factors for cancer with metals such as selenium (Se), zinc (Zn), arsenic (As), cadmium (Cd), and nickel (Ni), which are found naturally in the environment. Human exposure to these metals results from air, drinking water, and food [12, 13]. Other studies demonstrated that age-corrected breast cancer mortalities in different countries are inversely correlated with the dietary intake of Se and directly with the estimated intake of Cd, Zn, and chromium (Cr), suggesting that the anticarcinogenic properties of Se are counteracted by these elements [14]. As mentioned before, these metals can mimic the action of estrogen; the estrogenicity of various heavy metals was described in [15], for example, bis(tri-n-butyltin) > cadmium chloride > antimony chloride > barium chloride = chromium chloride > lithium hydroxide > sodium selenite = lead acetate > stannous chloride. 

While an association between exposure to metals and the risk of the lung, breast, colorectum, prostate, urinary bladder, and stomach cancers is discussed, it was demonstrated that breast cancer patients have abnormal levels of copper (Cu), Zn, Se, and Cd [16], Interestingly, other evidence shows an inverse association between Se exposure and prostate cancer and lung cancer risk. There is also some evidence for an inverse association between Zn and breast cancer, while there is no association between exposure to Se and the risk of breast, colorectal, and stomach cancer and between Zn and the risk to develop prostate cancer [12]. Nevertheless, positive associations of breast cancer with Zn, iron, and calcium, but little association with Se, have been reported in [17].

In particular, a study of [16] showed that the genetic instability found in stage I breast cancer patients (frequency of micronucleated lymphocytes) was related with the blood levels of Cu, Zn, Se, and Cd. The authors found that the level of Cu, Zn, and Se was significantly lower in breast cancer patients, as compared to controls while the level of Cd was significantly higher in these patients. In breast cancer patients, the frequency of micronucleated lymphocytes showed complex associations with different concentrations of these elements. High Cd, low Zn, low Se, and both high and low Cu levels increased the micronucleus formation in lymphocytes. A similar correlation was found in the control group only in relation to high Se and Cd levels [16].

## 3. Arsenic

Arsenic (As) exposure constitutes one of the most widespread environmental carcinogens and is associated with increased risk of different types of cancers [18–20]. Arsenites are found in drinking water and are used in wood preservatives, insecticides, and herbicides. Epidemiological evidence has associated exposure to As in drinking water with an increased incidence of human cancers in the skin, bladder, liver, kidney, and lung [21, 22], and low concentrations of As_2_O_3_ induce carcinogenesis after long-term exposure [23].

Nonetheless, arsenic trioxide (As_2_O_3_) is also a component of traditional Chinese medicine [24]. In clinics it is successfully used to treat hematologic malignancies. However, the antitumor effects could not be replicated for solid tumors [11, 23, 25], although *in vitro* As_2_O_3_ induces apoptosis in other solid cancer cell lines including breast cancer cells [19, 20, 23, 26]. In either application, the precise molecular mechanisms through which As_2_O_3_ induces cell cycle arrest and apoptosis in solid tumors have not been fully understood [24]. Hopefully, new insights into how As_2_O_3_ binds to specific receptors and how they trigger signaling pathways might facilitate As_2_O_3_-based anticancer strategies and/or combination therapies in order to treat solid tumors [18, 24]. 

A large body of evidence indicates that arsenic compounds induce cell death in breast cancer cells and the induction of this effect is a possible endorsement for the treatment of breast cancer. For example, sodium arsenite mimics the effects of estradiol and induces cell proliferation in the estrogen-responsive breast cancer cell line MCF-7 while the S-phase recruitment was increased [22]. Interestingly, regarding the cell proliferation, a paradox effect was observed: lower concentrations (<5 *μ*M) of sodium arsenite induced cell proliferation while higher concentrations (>5 *μ*M) or longer treatment periods induced apoptosis [21]. In addition, As also influences the enzymes participating in the folate cycle [21]. 

Other studies indicate that arsenite, in environmental relevant concentrations (5 *μ*M/0.65 mg/L), is able to induce both replication-dependent DNA double-strand breaks and homologous recombination. Double-strand break formation was replication dependent and probably the result of conversion of a DNA single-strand break into double-strand breaks [18]. In addition, low arsenite concentrations (0.5–5 *μ*M) induce ROS production and ROS-related depolarization of the mitochondrial membrane, suggesting that mitochondria play an important role in the oxidative effects of As. In addition, when ROS-mediated DNA damage is measured by the presence of 8-OHdG DNA adducts in their nuclei, I*κ*B phosphorylation, NF-*κ*B activation, and increases in c-Myc and HO-1 protein levels were also observed. Therefore, these factors might play a relevant role in the arsenite-induced MCF-7 cell recruitment into the S-phase of the cell cycle and cell proliferation observed [22]. Nonetheless, the authors conclude that arsenite activates several pathways involved in MCF-7 cell proliferation, and, therefore, arsenite exposure may pose a risk for breast cancer in exposed populations. 

Additionally it was found that, in MCF-7 breast cancer cells, As_2_O_3_ treatment changes the expression level of several genes that are involved in cell cycle regulation, signal transduction, and apoptosis. Important targets are represented by proteins which inhibit the cell cycle like p21 and p27. Liu et al. [23] and Wang et al. [24] showed that after 24 h exposure to As_2_O_3_ (0.01–1 *μ*M) cell proliferation significantly increased and a progression from the G1 to S/G2 phases occurred in the nontumorigenic MCF10A breast epithelial cell line. Several cell-cycle-associated genes were increased significantly, for example, cell division cycle 6 (CDC6) and cyclin D1 (CCND1) which are closely related to cell cycle progression from G1 to S phase. In addition, the production of ROS was elevated, while activation of p38 MAPK, Akt, and ERK1/2 pathways was observed [23]. Arsenite also increases in MT1/2 and c-Myc protein levels concentration dependently [21]. 

For treatment of solid tumors a novel nanoparticulate formulation of As_2_O_3_ encapsulated in liposomal vesicles named “nanobins” [NB(Ni, As)] was synthesized in order to improve the therapeutic efficiency against breast carcinomas. The NB (Ni, As) agent was less cytotoxic *in vitro* than As_2_O_3_, and *in vivo* NB (Ni, As) dramatically improved the therapeutic efficacy of As_2_O_3_. These effects are possibly due to a reduced plasma clearance, an enhanced tumor uptake, and an induction of tumor cell apoptosis [25].

## 4. Cadmium

Cadmium (Cd) is a nonessential metal that is dispersed throughout the environment [27, 28]. It has been categorized as a human carcinogen by the US Environmental Protection Agency. Primary exposure sources include food and tobacco smoke [8, 9, 27, 29]. Cadmium is a ubiquitous carcinogenic pollutant and has multiple biological effects, and exposure is correlated with the occurrence of breast cancer in some US regional case-control studies [2, 9, 10, 29]. 

Gallagher et al. [2] as well as McElroy et al. [9] observed a significant trend of an increased risk of breast cancer by elevated urinary cadmium concentrations, but the mechanisms of action of cadmium remain unclear [8, 30]. Cd affects multiple cellular processes, including cell proliferation, differentiation, and apoptosis [8]. Cd functions also as an endocrine disruptor, which stimulates estrogen-receptor-*α* (ER-*α*) activity and promotes uterine and mammary gland growth in and abolishes the cancer-protecting effects of Se in female inbred C3H mice carrying murine mammary tumor virus [14].

Cd modulates gene expression, affects the pattern of transcriptional activity, and, therefore, changes intracellular signals [31]. The modification of gene expression in MCF7 cells is blocked by antiestrogens. Therefore, these effects could be mediated by ER-*α* [29, 30]. In estrogen-responsive breast cancer cell lines, Cd stimulates proliferation and also activates the estrogen receptor independent of estradiol [28–30]. Cd activates extracellular regulated kinases, erk-1 and -2 in both ER-positive and ER-negative human breast cancer cells. High Cd concentrations from 50 to 500 nM induced a proliferative response SKBR3 cells, increased intracellular cAMP levels. Cd treatment activates raf-1, mitogen-activated protein kinase kinase, mek-1, extracellular signal-regulated kinases, erk-1/2, ribosomal S6 kinase, rsk, and E-26 like protein kinase, elk [28].

ER-*α* is required for both Cd-induced cell growth and modulation of gene expression. ER-*α* translocates to the nucleus in response to Cd exposure and potentiates the interaction between ER-*α* and c-Jun and enhances recruitment of this transcription factor complex to the proximal promoters of cyclin D1 and c-myc, increasing the mRNA expression [8]. Additionally, Casano and colleagues in 2010 [31] confirmed that treatment of breast cancer cells with 5 *μ*M CdCl_2_ induces a diversified modulation of the transcription patterns of p38, while [30] Sun and coworkers (2007) showed that treatment of MCF-7 cells with Cd resulted in induction of Hsp22. Cd increases breast cancer cell proliferation *in vitro* by stimulating Akt, ERK1/2, and PDGFR*α* kinases activity likely by activating c-fos, c-jun, and PDGFA by an ER-*α*-dependent mechanism [32].

In chronic Cd exposure (over 40 weeks) of the human breast epithelial cell line MCF-10A, secretion of matrix metalloproteinase-9 increased, followed by a loss of contact inhibition, increased colony formation, and increasing invasion. Furthermore, inoculation of Cd-treated cells into mice produced invasive, metastatic anaplastic carcinoma. Additionally, breast stem cell markers CK5 and p63 were found overexpressed indicating persistent proliferation, global DNA hypomethylation, and c-myc and k-ras overexpression [10]. Exposure of breast cancer cells to “subtoxic” levels of Cd significantly inhibited the angiogenic potential of the breast cancer cell line, suggesting the possibility that Cd might negatively regulate the production of proangiogenic factors in breast cancer cells. 

Interestingly, melatonin prevents the Cd-induced growth of synchronized MCF7 breast cancer cells. Melatonin is a specific inhibitor of Cd-induced ER-*α*-mediated transcription, inhibits MCF7 cell growth induced by Cd, and regulates Cd-induced transcription in both ERE and AP1 pathways. Overall, the antiestrogenic properties of melatonin might be a valuable tool in breast cancer therapies [29].

In summary, Cd might exert a paradoxical effect in breast cancer: on the one hand, it could promote carcinogenesis, and, on the other hand, it could delay the onset of tumors by inhibiting breast cancer cell-induced angiogenesis [27].

## 5. Gold

Gold nanoparticles (GNPs) are regarded as a possible delivery vehicle for anticancer drugs and seem to have a great potential to be used in clinics. In breast cancer MDA-MB-231 cells the group of Jain [33] assessed the cellular uptake, intracellular localization, and cytotoxicity of GNPs. When GNPs were taken up, nanoparticles accumulated in cytoplasmic lysosomes. However, the GNP exposure did not increase radiation-induced double-strand breaks formation and did not inhibit DNA repair; but GNP chemosensitization was observed in MDA-MB-231 cells treated with bleomycin [33].

For some time it was assumed that the GNPs are but recent studies showed that this is not the case since it was demonstrated that they cause oxidative stress and even cell death, suggesting a possible biological mechanism for sensitization [33]. Using syngeneic mouse and human xenograft models of triple-negative breast cancer, Atkinson and his group demonstrated that local hyperthermia generated by gold nanoshells plus radiation eliminates radioresistant breast cancer stem cells [6]. Another study by Day et al. [34] describes the possibility of using near-infrared resonant gold-gold sulfide nanoparticles as dual contrast and therapeutic agents for cancer management via multiphoton microscopy followed by higher intensity photoablation which can be utilized to visualize cancerous cells *in vitro*. When conjugated with anti-HER2 antibodies, these nanoparticles specifically bind SK-BR-3 breast carcinoma cells that overexpress the HER2 receptor, enabling the cells to be imaged via multiphoton microscopy [34].

## 6. Platinum

Cisplatin is a first choice chemotherapeutic drug for different types of cancer. Although there is increasing evidence that breast cancers are sensitive to cisplatin, its clinical success is often compromised due to dose-limiting nephrotoxicity and the development of drug resistance. To overcome these limitations, other platinum derivatives have been developed for the treatment of breast cancer, and several of them are still tested in clinical trials. In addition multiple drug combination therapies (with include cisplatin) have been employed [11, 35].

Multiple cellular effects have been described for cisplatin (for review see [11]). Recently it was demonstrated that cisplatin increases the intracellular calcium concentration dependently, and this increase of the intracellular calcium signal is directly related to cytotoxicity [36]. This is in agreement with similar results which were found earlier with other cancer cell lines [37].

Regarding the molecular effects triggered by cisplatin in breast cancer cells, Wong et al. [35], showed that inhibition of the mTOR, TGFbetaRI, NF*κ*B, PI3K/AKT, and MAPK pathways sensitized basal-like MDA-MB-468 cells to cisplatin treatment. Nevertheless, the combination of the mTOR inhibitor rapamycin and cisplatin generated significant drug synergism in basal-like MDA-MB-468, MDA-MB-231, and HCC1937 cells but not in luminal-like T47D or MCF-7 cells. The synergistic effect of rapamycin plus cisplatin was mediated by the induction of p73. The authors conclude that a combination therapy with mTOR inhibitors and cisplatin could be a useful therapeutic strategy for the treatment of basal-like breast cancers.

A combination of gemcitabine and cisplatin was tested in metastatic breast cancer and was successful in phase II trials. This suggests that the combination of gemcitabine and cisplatin is a safe and tolerable regimen and useful as second-line combination for patients with anthracycline- and taxane-pretreated MBC. It is mostly used as a salvage regimen for progressive disease refractory to anthracyclines and taxanes and when liver dysfunction secondary to liver metastasis precludes these drugs [26, 38, 39]. 

It is also discussed whether a combination of cisplatin and TRAIL has the potential to improve the therapeutic outcome in triple-negative breast cancer (TNBC) patients. This approach was tested *in vitro* on normal and triple-negative breast cancer (TNBC cells) by Xu and coworkers [40]. Indeed, this combination significantly enhanced cell death in TNBC cell lines and inhibited the expression of EGFR, p63, survivin, Bcl-2, and Bcl-xL. Specific inhibition of EGFR and/or p63 protein in TNBC cells was observed while survivin played an important role in cisplatin plus TRAIL-induced apoptosis in TNBC cells. *In vivo *experiments resulted in a significant inhibition of CRL2335 xenograft tumors compared to untreated control tumors [40].

It was speculated that whole body thermal therapy would boost the efficacy of oxaliplatin chemotherapy with reduced toxicity. Indeed, elevating the temperature reduced the IC_50_ of oxaliplatin in MTLn3 cells, while the cellular uptake of platinum and platinum adducts increased. *In vivo*, 50% of all oxaliplatin treated rats 24 h before thermal therapy were immunologically cured; in 11% their primary tumor regressed but ultimately succumbed to metastases, and 17% experienced a limited response with increased survival. In uncured animals, the thermo-chemo-therapy had a delayed incidence and slowed growth of metastases [41].

The inhibitory activity of different anticancer metal complexes based on platinum, ruthenium, and gold metal ions was evaluated on the zinc-finger protein PARP-1, either purified or directly on protein extracts from human breast cancer MCF7 cells. The results by Mendes and coworkers [42] support a model whereby displacement of zinc from the PARP-1 zinc finger by other metal ions leads to decreased PARP-1 activity. *In vitro* combination on different cancer cell lines, including MCF7, showed synergistic effects [42]. 

New platinum compounds are yet to be studied for their potential to be used in anticancer treatment. A series of seven platinum (II) cyclobutane-1,1-dicarboxylato (cbdc) complexes {[Pt(cbdc)(L(n))(2)], 1–7}, derived from carboplatin were studied for their *in vitro* cytotoxicity activity against breast adenocarcinoma cells (MCF-7) and were found to be cytotoxic [43]. Recently, Paraskar and colleagues [44] reported a novel polymer, glucosamine-functionalized polyisobutylene-maleic acid, where platinum (Pt) can be complexed to the monomeric units. This complex self-assembles to a nanoparticle, which releases cisplatin in a pH-dependent manner. Those nanoparticles are rapidly internalized into the endolysosomal compartment of cancer cells and exhibited a significantly improved antitumor efficacy in breast cancers. Furthermore, the nanoparticle treatment resulted in a reduced systemic and nephrotoxicity, which was due to a decreased distribution of platinum to the kidney [44]. The *in vitro* antitumor activity of the [Pt(ox)(L(n))(2)] (1–7) and [Pd(ox)(L(n))(2)] (8–14) oxalato complexes involving N6-benzyl-9-isopropyladenine-based N-donor carrier ligands (L(n)) against breast adenocarcinoma (MCF7) were studied by Paraskar and coworkers [44]. This group found the tested complexes to be more cytotoxic compared to cisplatin, but they were non-hepatotoxic [44].

In MCF-7 cells [Pt(O,O′-acac)(*γ*-acac)(DMS)] had toxic effects at high concentrations, while subcytotoxic concentrations induced anoikis and decreased cell migration. This compound altered [Ca^2+^]_i_ homeostasis and triggered apoptosis. When cells were stimulated with ATP, the changes in Ca^2+^ levels caused by purinergic stimulation were altered due to decreased PMCA activity and due to the closure of Ca^2+^ channels opened by purinergic receptors. Conversely, [Pt(O,O′-acac)(*γ*-acac)(DMS)] did not affect the store-operated Ca^2+^ channels opened by thapsigargin or by ATP, but it provoked the activation of PKC-*α* and the production of ROS that were responsible for the Ca^2+^ permeability and PMCA activity decrease [45].

## 7. Lead

Breast cancer incidence in women has been related to industrialization suggesting that the associated widespread contamination of the soil, air, and the water by lead (Pb) and other industrial metals is a major risk factor. Due to its wide use, Pb is of particular concern. In levels as low as 0.5 ppm Pb (in drinking water), it promotes the development of mammary murine tumors in virus-infected female C3H mice [46]. It also accelerates tumor growth rates. Higher levels of Pb were found in blood and head hair samples of newly diagnosed patients with breast cancer, all with an infiltrating ductal carcinoma [46]. The Pb levels in the hair samples were directly correlated with the volumes of the tumors [46]. The same researchers also found evidence that Pb and other metals also interact with iodine, a vitally important essential trace element that most likely protects against breast cancer development [46].

On the other side, some new metal-organic lead structures have been developed over the last years, which actually exhibit cytostatic properties. However, the efficiency of such chemotherapeutics in the treatment of tumors might be limited by their low therapeutic index due to their short half-life, lack of tumor selectivity, and associated side effects [46].

## 8. Cymantrene-Peptide Conjugates

Cymantrene (CpMn(CO_3_)) is a robust organometallic group, which is stable in air and water. In experiments done by Splith and coworkers [47], cymantrene derivatives were attached to the cell-penetrating peptide sC18 which acted as a transporter for the metal moiety. This group characterized the conjugates for their cytotoxic activity on human breast adenocarcinoma cells (MCF-7) and human colon carcinoma cells (HT-29). These researchers found that bioconjugates bearing two cymantrene groups were more active than the monofunctionalized ones and that, by the introduction of a cathepsin B cleavage site next to the organometallic group, the biologic activity was increased [47].

## 9. Selenium

The role of Se as a potential cancer chemopreventive and chemotherapeutic agent has been supported by epidemiological, preclinical, and clinical studies [48–50]. Se levels in hair and blood were inversely correlated with tumor volumes, which are consistent with the antiproliferative effects of Se [46].

While cell apoptosis is a critical mechanism mediating the anticancer activity of Se, the underlying molecular mechanisms still remain elusive [48]. The anticancer properties of Se might be due to the fact that it partially protects against oxidative stress [51]. The same group also assessed whether supplementation of BRCA1 mutation carriers with Se has a beneficial effect to oxidative stress/DNA damage since Se supplementation in patients may result in reduction of oxidative DNA damage. They found that BRCA1 deficiency contributes to 8-oxodG accumulation in cellular DNA, which in turn is a factor responsible for cancer development in women [51].

Se compounds modify gene expression. When breast epithelial cells (MCF-10A) were exposed to 100 nM sodium Se or high-Se serum, the expression of 560 genes including 60 associated with the cell cycle were affected. The group of Hawkes et al. [52] describes that selenoprotein W (SEPW1) was the only selenoprotein increased by both sodium selenite (specific) and high-Se serum (physiologic). SEPW1 small interfering RNA inhibited G1-phase progression and increased G1-phase gene transcripts while decreasing S-phase and G2/M phase gene transcripts, indicating that the cell cycle was interrupted at the G1/S transition. SEPW1 mRNA levels were maximal during G1 phase, dropped after the G1/S transition, and increased again after G2/M phase. SEPW1-underexpressing prostate cells had increased mRNA for BCL2, which can induce a G1 arrest and decreased mRNA for RBBP8 and KPNA2, which modulate the Rb/p53 checkpoint pathway. Altogether, these results suggest that SEPW1 and the G1/S transition are physiological targets of Se in breast and prostate epithelial cells [52]. 

Selenocysteine (SeC), a naturally occurring selenoamino acid, induces a caspase-independent apoptosis in MCF-7 breast carcinoma cells, accompanied by poly (ADP-ribose) polymerase (PARP) cleavage, caspase activation, DNA fragmentation, phosphatidylserine exposure, and nuclear condensation. Moreover, SeC induced a loss of the mitochondrial membrane potential (Delta Psi (m)) involving the expression and phosphorylation of Bcl-2 family members. Loss of Delta Psi (m) induced the mitochondrial release of cytochrome C and apoptosis-inducing factor followed by chromatin condensation and DNA fragmentation. MCF-7 cells exposed to SeC showed an increase in total p53 and phosphorylated p53 prior to mitochondrial dysfunction. Silencing and attenuating of p53 activation partially suppressed SeC-induced cell apoptosis. Furthermore, generation of reactive oxygen species and the induction of DNA strand breaks were found. Therefore, SeC could be a promising anticancer compound, which induces MCF-7 cell apoptosis by activating the ROS-mediated mitochondrial pathway and p53 phosphorylation [48]. 

Se might be beneficial in combination with other drugs in adjuvant therapy. Therefore, the combination of anticancer drugs with Se combinations was investigated. Li et al. [53, 54] investigated the therapeutic effect of methylselenocysteine (MSC) combined with tamoxifen in MCF-7 breast cancer xenograft. Indeed, treatment with tamoxifen together with MSC synergistically inhibited tumor growth compared to MSC alone and tamoxifen alone. MSC alone or MSC + tamoxifen significantly reduced ER*α*, PR and cyclin D1, Ki67 index, and microvessel density while increasing apoptosis in tumor tissues. These findings demonstrate a synergistic growth inhibition of ER*α*-positive breast cancer xenografts for a combination of tamoxifen with organic selenium compounds [53, 54].

The group of Li showed that combining doxorubicin with selenium resulted in an enhancement of apoptosis in MCF-7 human breast cancer cells [55, 56]. They found that mitochondrial activation of caspase-9 is in part responsible for the synergy; while the death receptor pathway was involved in the activation of caspase-8. On the other hand, Se increased the expression of FADD, which is responsible for recruitment of caspase-8 to the Fas oligomer. Therefore, doxorubicin and selenium cooperatively activate Fas signaling by targeting key regulatory steps [56]. Se was capable of depressing doxorubicin-induced Akt phosphorylation, important in mediating the synergy between Se and doxorubicin. Se reduced the abundance of phospho-GSK3*β* induced by doxorubicin, whereas chemical inhibition of GSK3*β* activity muted the apoptotic response to the Se/doxorubicin combination. Se increased the transactivation activity of FOXO3A [55].

## 10. Conclusion

Metals and metal compounds interfere with breast cancer in multiple ways. On the one side, they are an important risk factor for the development of breast cancer, while on the other side their cytotoxicity might have also beneficial effects in inducing apoptosis and cytotoxicity in breast cancer cells. To highlight this delicate balance and to understand under which circumstances specifically cancer cells could be targeted by metals and their compounds, further research is needed.
